# Analysis of physical activity levels and influencing factors in cancer survivors after pancreaticoduodenectomy

**DOI:** 10.3389/fonc.2024.1428884

**Published:** 2025-01-16

**Authors:** Qiuju Tian, Meiqin Xue, Leying Chen, Min Zhang, Weiyi Zhu, Beiwen Wu

**Affiliations:** ^1^ Gastrointestinal Surgery Department, Ruijin Hospital, Shanghai Jiaotong University School of Medicine, Shanghai, China; ^2^ Nursing Department, Ruijin Hospital, Shanghai Jiaotong University School of Medicine, Shanghai, China; ^3^ Pancreatic Surgery Department, Ruijin Hospital, Shanghai Jiaotong University School of Medicine, Shanghai, China

**Keywords:** cancer survivors, physical activity, pancreatic cancer, pancreaticoduodenectomy, pancreatic fistula

## Abstract

**Introduction:**

Physical activity is becoming more important in cancer patient care. However, there are limited studies investigating physical activity levels in cancer survivors after pancreaticoduodenectomy. This study aims to assess the present status of physical activity levels in cancer survivors after pancreaticoduodenectomy and whether perioperative metrics and length of follow-up have an impact on physical activity levels in survivorship.

**Methods:**

This is a cross-sectional study. The study included cancer survivors who were treated at a tertiary general teaching hospital for pancreaticoduodenectomy from December 2019 to January 2022 following surgery. We quantified physical activity frequency, duration, and intensity using the International Physical Activity Questionnaire-Short Form. Patient demographic and clinical characteristics were obtained via an electronic medical record system. Postoperative complication data were obtained from our survival cohort. Variables univariately associated with the physical activity level at an alpha level of less than 0.1 were included in the logistic regression analysis of factors influencing physical activity in cancer survivors after pancreaticoduodenectomy.

**Results:**

A total of 223 patients who met the eligibility criteria completed a telephone survey. The main form of physical exercise is walking, 69.5% of participants’ physical activity belongs to the active category, but only 16.6% of participants met the aerobic guideline. Logistic regression showed that cancer survivors without pancreatic fistula were 2.453 times more likely to perform active physical activity in survival than those with pancreatic leakage (*p* = 0.041). For a one-unit increase in operation duration, there is approximately a 0.5% reduction in the level of active physical activity participation among cancer survivors after pancreaticoduodenectomy (*p* = 0.015). For each unit increase in follow-up time, post-pancreaticoduodenectomy patients were 1.046 times more likely to participate in active physical activity (*p* = 0.030).

**Conclusion:**

Although half of the cancer survivors after pancreaticoduodenectomy experienced active physical activity, only a small percentage of individuals met the guideline-recommended level of aerobic exercise. More physical activity support should be provided to cancer survivors after pancreaticoduodenectomy. Moreover, operation duration, postoperative pancreatic fistula, and follow-up time should be taken into consideration when giving exercise instructions to postoperative survivors of pancreaticoduodenectomy.

## Introduction

Pancreatic cancer remains one of the most malignant solid tumors with a 5-year survival of 5%–12% in all stages (1). Pancreaticoduodenectomy, the so-called “Whipple operation”, is a classic surgical approach to treating tumors in the head or body of the pancreas and ampulla of Vater. Because of this surgical technique refinement, combined with adjuvant and neoadjuvant therapy improvement, prolonged survival in patients with resectable pancreatic cancer has been achieved (2). Clinical trials utilizing adjuvant chemotherapy and chemoradiotherapy resulted in steady improvements in median overall survival with dramatic improvements to 46.5 months (3) and 54.5 months (4), respectively. However, cancer survivors tend to experience physical and psychological deconditioning after cancer therapy (5). Thus, it is important to pay attention to the quality of life among survivors.

Physical activity is becoming more important in cancer patient care. International guidelines and recommendations highlight the importance of physical activity during and after cancer treatment (6–9). In addition, some systematic reviews and scoping reviews confirmed that physical activity in patients with pancreatic cancer is feasible and can improve their muscle strength, cancer-related fatigue, and quality of life (10–12). Recently, a randomized controlled study with a 12-month follow-up shows that supervised physiotherapy or prescribed home-based exercise after pancreatic cancer resection is safe and feasible, and should be proposed and started as soon as possible to improve certain aspects of quality of life (13). The intensity of physical activity in humans is expressed in metabolic equivalents of task (METs), defined as the energy it takes for an adult to sit quietly and is roughly equivalent to the expenditure of one kilocalorie per kilogram of body weight per hour (14). Cancer survivors are encouraged to do moderate-intensity aerobic training at least three times per week, for at least 30 min for 8 to 12 weeks to address health-related outcomes after cancer treatment. However, there are limited studies investigating physical activity levels in survivors of pancreatic cancer after surgery (15). Parker et al. found that 15.6%–21.4% pancreatic survivors (*N* = 262) met the American College of Sports Medicine (ACSM) guideline, and age, body mass index (BMI), a history of pancreatoduodenectomy or total pancreatectomy, barrier self-efficacy, and behavioral regulation were associated physical activity (15). In addition, a telephone survey found that barriers to participation included older age and physical, personal, and emotional problems in pancreatic cancer survivors (*N* = 50) (16). To our knowledge, there are no data on physical activity levels in Chinese cancer survivors after pancreaticoduodenectomy.

Therefore, we conducted the present study to quantify physical activity among survivors following pancreaticoduodenectomy for pancreatic cancer or adenocarcinoma of the ampulla of Vater, or duodenal cancer or bile duct cancer, and analyze whether perioperative factors have an effect on the physical activity level in survivors.

## Materials and methods

### Study design and inclusion criteria

This was a telephone survey study of cancer survivors previously treated at an academic hospital for pancreaticoduodenectomy from December 2019 to January 2022. The telephone survey took approximately 10 min for participants to complete. Prospective patients were identified from the survival cohort study, and patient information, including age, gender, ethnicity, marital status, education level, employment, religion, tobacco usage, alcohol usage, comorbid condition, Nutrition Risk Screening 2002 (NRS2002) score, histopathologic diagnosis, operation type, time since resection, and postoperative complication were obtained via an electronic medical record system. The definition and grading system of postoperative pancreatic fistula are according to the International Study Group of Pancreatic Fistula (ISGPF) in 2016. A pancreatic fistula is defined as the detection of fluid output from surgically placed abdominal drains containing amylase levels greater than three times the upper limit of normal serum values more than 3 days postoperatively. A clinical grading system categorizes postoperative pancreatic fistula into biochemical leak, grade B, and grade C based on the severity of complications. Biochemical leaks are not included in the statistics for pancreatic fistula complication in this study (17). Inclusion criteria included the following: (1) pancreatic cancer or duodenal cancer or bile duct cancer or adenocarcinoma of the ampulla of Vater; (2) subjects undergoing pancreaticoduodenectomy; (3) age greater than or equal to 18 years old; and (4) consent to participate. The studies involving humans were approved by the Ruijin Hospital Ethic Committee. The studies were conducted in accordance with the local legislation and institutional requirements.

We use the Chinese version of the International Physical Activity Questionnaire-Short Form (IPAQ-SF) to survey the usual 7 days of physical activity in October 2023, with a test–retest reliability of 0.626–0.887 and a criterion validity of 0.60 (18). IPAQ-SF assesses the types of intensity of physical activity and sitting time that people do as part of their daily lives, which are considered to estimate total physical activity in METs-min/week. The official IPAQ guidelines assigned the physical activity intensity of 3.3, 4.0, and 8.0 METs for walking, moderate physical activity, and vigorous physical activity, respectively. Weekly minutes of moderate and vigorous aerobic exercise were added to compute moderate to vigorous exercise volume. Estimates of exercise guideline concordance were based on the ACSM exercise guidelines for cancer survivors, and participants were categorized as meeting the aerobic exercise guideline if they reported at least 90 min per week of moderate to vigorous exercise volume.

The survivors were divided into three categories. Category 1, named inactive, represents the lowest level of physical activity. Individuals who do not meet the criteria for categories 2 or 3 are deemed to have “insufficient activity”. Meeting any of the following three criteria could be classified as minimally active (category 2): (a) 3 days of vigorous activity of at least 20 min/day; (b) 5 days of moderate-intensity activity or walking >30 min/day and for >10 min at a time; or (c) 5 days of any combination of walking, moderate-intensity or vigorous-intensity activities achieving at least 600 MET-min/week. Meeting either of two criteria will be categorized as high-intensity physical activity (HEPA) active (category 3): (a) vigorous-intensity activity on >3 days/week and accumulating at least 1,500 MET-min/week; or (b) >5 days of any combination of walking, moderate-intensity, or vigorous-intensity activities achieving at least 3,000 MET-min/week (19).

### Sample size

The sample size was calculated based on the sample requirement for regression analysis, which was at least 10–20 times the number of independent variables. In the present study, the number of independent variables was 6. Thus, the sample size was 120.

### Statistical analysis

Clinicodemographic characteristics and survey data were summarized as categorical variables with numbers and percentages, or non-normally distributed continuous variables with median and 25th percentile (P25) and 75th percentile (P75). Category 2 and category 3 are added as active physical activity group. The χ² test for categorical variables was used to compare the differences in the inactive and active group for categorical variables, and the non-parametric test for two independent samples was used to compare the differences in two groups for non-normally distributed continuous variables. Variables univariately associated with the physical activity level at an alpha level less than 0.1 were included in the logistic regression analysis of factors influencing physical activity in cancer survivors after pancreaticoduodenectomy. All analyses were conducted utilizing SPSS 20.0. The significance level was set at *p*<0.05 in all analyses.

## Results

### Descriptive characteristics of the participants

**Figure 1:**
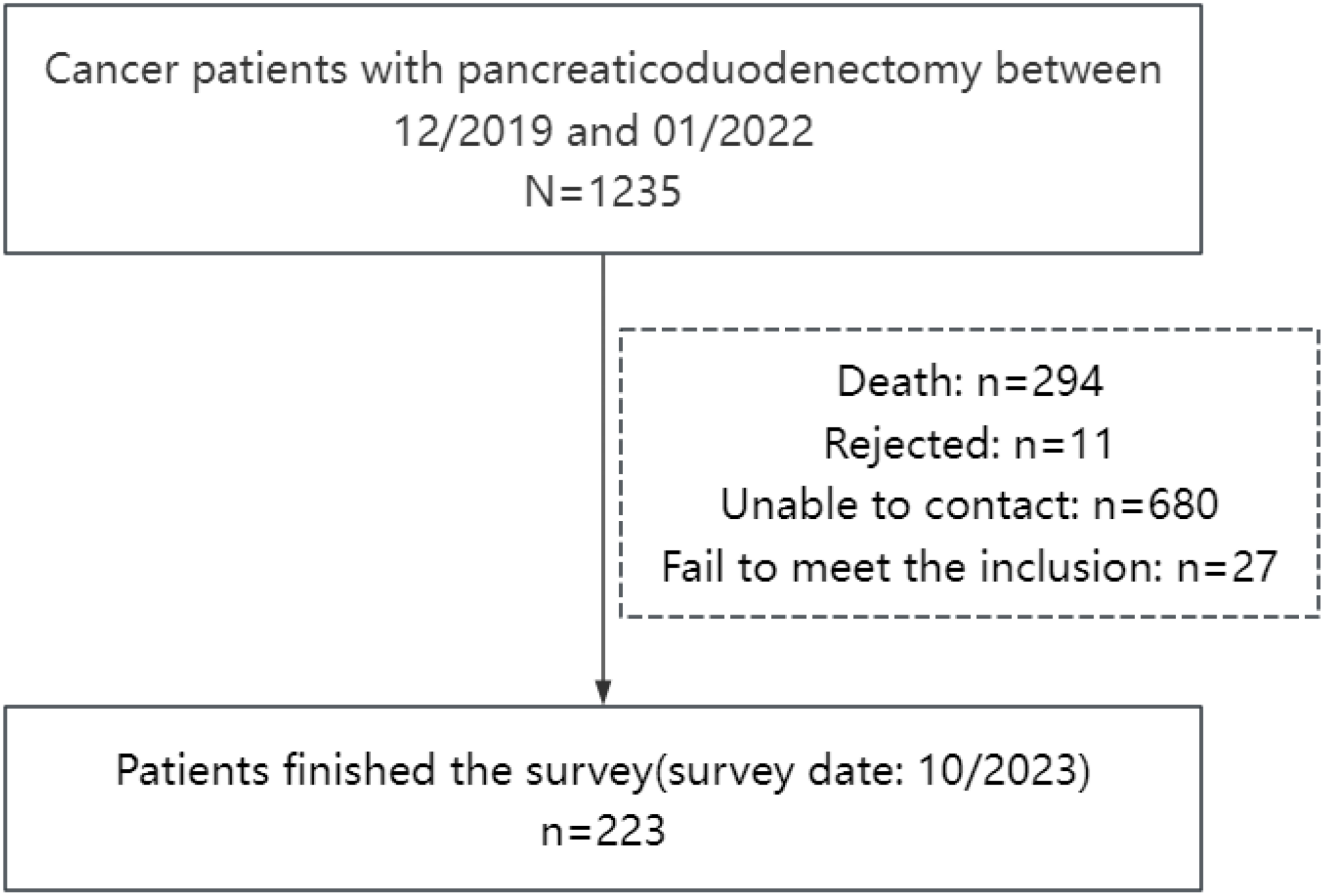
Study flowchart.

There were 1,235 patients undergoing pancreaticoduodenectomy between December 2019 and January 2022. A total of 223 patients who met the eligibility criteria completed a telephone survey with a median of 28 (22, 37) months following surgery (**Figure 1**). The shortest time from tumor resection to completion of follow-up in this study was 16 months, and the longest was 45 months. The average age of non-responders to the interview was 63 (55,69) years old, and 55.91% of them were male. The average age of the participants was 63 (56,69) years old, and 57.4% were male. There was no significant difference in age (*Z* = −0.128, *p* = 0.828) and gender (χ² = 0.153, *p* = 0.696) between the non-responders to the interview and participants. Approximately 74.9% of participants were diagnosed with pancreatic cancer. There were 145 (65%) patients with secondary and below education level. Approximately 53.8% of patients were at stage I of the disease and 17% were at nutritional risk. The average hospital stay time was 24 (19, 32) days. Clinicodemographic data for the participants are reported in **Table 1**. Approximately 22% of participants had postoperative complications, including infection (45), pancreatic fistula (27), biliary fistula (13), gastroparesis (9), gastrointestinal bleeding (6), abdominal hemorrhage (6), gastrointestinal fistula (2), and others (10). All cases of pancreatic fistulas encountered in this study were classified as Grade B.

**Table 1:** Demographic and clinical characteristics of the participants in the study sample.

Variables	Values
Age, *n* (%)	
<60 years	78 (35.0)
≥60 years	145 (65.0)
Gender, *n* (%)	
Male	128 (57.4)
Female	95 (42.6)
Marital status, *n* (%)	
Married	216 (96.9)
Single/widowed/divorced	7 (3.1)
Education, *n* (%)	
Secondary and below	145 (65.0)
High school	17 (7.6)
Graduate and above	61 (27.4)
Ethnicity, *n* (%)	
Han	220 (98.7)
Others	3 (1.3)
Employment, *n* (%)	
Employed	54 (24.2)
Unemployed	169 (75.8)
Religion, *n* (%)	
No	214 (96.0)
Yes	9 (4.0)
Tobacco usage, *n* (%)	
No	188 (84.3)
Yes	35 (15.7)
Alcohol usage, *n* (%)	
No	194 (87.0)
Yes	29 (13.0)
Comorbid condition, *n* (%)	
No	107 (48.0)
Yes	116 (52.0)
Tumor type, *n* (%)	
Adenocarcinoma	138 (59.5)
Papilloma	34 (14.7)
Neuroendocrine tumor	20 (8.6)
Others	40 (17.2)
Tumor location, *n* (%)	
Pancreas	167 (74.9)
Duodenum	24 (10.8)
Bile duct	15 (6.7)
Ampulla of Vater	17 (7.6)
Disease stage at initial diagnosis, *n* (%)	
I	120 (53.8)
II	76 (34.1)
III	21 (9.4)
IV	6 (2.7)
BMI, *n* (%)	
<18.5 kg/m^2^	6 (2.7)
18.5–23.9 kg/m^2^	119 (53.4)
≥24 kg/m^2^	98 (43.9)
NRS2002 score, *n* (%)	
<3	185 (83.0)
≥3	38 (17.0)
Minimal invasiveness	
No	157 (70.4)
Yes	66 (29.6)
Postoperative complications, *n* (%)	
0	135 (60.5)
1	64 (28.7)
2	19 (8.5)
3	5 (2.2)
Hospital stay, median (25%,75%)	24 (19, 32)
Barthel index, median (25%,75%)	100 (100, 100)
Months between surgery and survey completion, median (25%,75%)	28 (22, 37)

NRS 2002, Nutrition Risk Screening 2002; BMI, body mass index.

As presented in **Table 2**, the average sedentary time per day was up to 7 (5, 8) h, and 69.5% of participants’ physical activity belongs to the active category. Notably, median METs-min/week in the inactive, minimally active, and HEPA active category was 238 (0, 495), 1,109 (693, 1,386), and 4,452 (3,066, 6,132), respectively. Only 16.6% of participants met the ACSM aerobic guideline.

**Table 2:** Self-reported physical activity level according to IPAQ short form.

Variables	Values
Physical activity level, *n* (%)	
Category 1—Inactive	68 (30.5)
Category 2—Minimally active	140 (62.8)
Category 3—HEPA active	15 (6.7)
Sedentary time, hours/day, median (25%,75%)	7 (5, 8)
Met aerobatic guideline, *n* (%)	
Yes	37 (16.6)
No	186 (83.4)

IPAQ, International Physical Activity Questionnaire; HEPA, high-intensity physical activity.

### Factors influencing physical activity in patients after pancreaticoduodenectomy

In a univariate analysis (**Table 3**), we found that cancer survivors with preoperative nutritional risk, BMI < 18.5 kg/m^2^, longer operation duration, pancreatic leakage, abdominal bleeding, and shorter or longer months between surgery and survey completion were less likely to perform active physical activity after pancreaticoduodenectomy (all *p*<0.05).

**Table 3:** The comparison of physical activity level in different groups of demographic and clinic characteristics.

Variables	Inactive	Active	χ²/*Z*	*p*
Age, *n* (%)			0.721	0.396
<60 years	21 (26.9)	57 (73.1)		
≥60 years	47 (32.4)	98 (67.6)		
Gender, *n* (%)			0.763	0.462
Male	42 (32.8)	86 (67.2)		
Female	26 (27.4)	69 (72.6)		
Education, *n* (%)			1.604	0.448
Secondary and below	47 (32.4)	98 (67.6)		
High school	3 (17.6)	14 (82.4)		
Graduate and above	18 (29.5)	43 (70.5)		
Comorbid condition, *n* (%)			0.477	0.490
0	35 (32.7)	72 (67.3)		
1	33 (28.4)	83 (71.6)		
NRS2002 score, *n* (%)			4.385	0.036
<3	51 (27.6)	134 (72.4)		
≥3	17 (44.7)	21 (86.5)		
Cancer location			5.609	0.132
Pancreas	44 (26.3)	123 (73.7)		
Duodenum	10 (41.7)	14 (58.3)		
Bile duct	6 (40.0)	9 (60.0)		
Ampulla of Vater	8 (47.1)	9 (52.9)		
Complication, *n* (%)			0.120	0.729
No	40 (29.6)	95 (70.4)		
Yes	28 (31.8)	60 (68.2)		
Pancreatic leakage, *n* (%)			4.518	0.034
No	55 (28.1)	141 (71.9)		
Yes	13 (48.1)	14 (51.9)		
Abdominal bleeding, *n* (%)			3.807	0.051
No	64 (29.5)	153 (70.5)		
Yes	4 (66.7)	2 (33.3)		
Minimal invasiveness, *n* (%)			1.584	0.242
No	50 (31.8)	107 (68.2)		
Yes	18 (27.3)	48 (72.7)		
TNM stage, *n* (%)			0.798	0.850
I	39 (57.4)	81 (52.3)		
II	22 (32.4)	54 (34.8)		
III	5 (7.4)	16 (10.3)		
IV	2 (2.9)	4 (2.6)		
Operation duration, *n* (%)	330 (272.3, 360)	300 (240, 335)	−2.843	0.004
BMI, *n* (%)			7.070	0.029
<18.5 kg/m^2^	4 (66.7)	2 (33.3)		
18.5–23.9 kg/m^2^	29 (24.04)	90 (75.6)		
≥24 kg/m^2^	35 (35.7)	63 (64.3)		
Hospitalization	0 (0,1)	0 (0, 3)	<0.001	1.000
Hospital stay	25 (18.2, 40)	24 (20, 30)	−1.047	0.295
Months between surgery and survey	25 (20, 32.8)	29 (22, 38)	−2.767	0.006

NRS 2002, Nutrition Risk Screening 2002; BMI, body mass index.

As shown in **Table 4**, logistic regression analysis was used to identify perioperative factors influencing physical activity in patients after pancreaticoduodenectomy, where variables with a *p*-value less than 0.1 on univariate analysis were included in the multivariate analysis. The results indicate that patients without pancreatic fistula were 2.453 times more likely to perform active physical activity in survival than those with pancreatic leakage (*p* = 0.041). For one-unit increase in operation duration, there is approximately a 0.5% reduction in the level of active physical activity participation among cancer survivors after pancreaticoduodenectomy (*p* = 0.015). For each unit increase in follow-up time, cancer survivors after pancreaticoduodenectomy were 1.046 times more likely to participate in physical activity (*p* = 0.030).

**Table 4:** Logistic regression analysis of factors influencing physical activity in patients after pancreaticoduodenectomy.

Variables	β	SE	Wald χ²	95% CI	OR	*p*
NRS2002 score ≥3 vs. <3 (ref.)	0.397	0.427	0.865	0.644, 3.432	1.487	0.352
Pancreatic fistula Grade B vs. No (ref)	0.897	0.438	4.196	1.040, 5.789	2.453	0.041
Abdominal bleeding Yes vs. No (ref.)	−0.943	0.985	0.916	0.056, 2.687	0.389	0.339
Operation time	−0.005	0.002	5.917	0.99. 0.999	0.995	0.015
BMI 18.5–23.9 kg/m^2^	1.436	0.982	2.138	0.613, 28.843	4.205	0.144
BMI ≥24 kg/m^2^	0.973	0.988	0.970	0.382, 18.345	2.646	0.325
BMI <18.5 kg/m^2^ (ref.)						
Months between surgery and survey completion	0.045	0.021	4.731	1.004, 1.089	1.046	0.030

NRS 2002, Nutrition Risk Screening 2002; BMI, body mass index; SE, standard error; CI, confidence interval.

## Discussion

Physical activity is considered safe for adults living with cancer without contraindications. Yeo et al. found that a progressive post-resection walking program could significantly improve fatigue and health-related quality of life in patients with pancreas and periampullary cancer (20). Kurz et al. discover that aerobic exercise slows pancreatic cancer growth in mice through activation of the immune system, particularly CD8+ T cells. The beneficial effects of exercise can also be mimicked by treatment with an IL-15 super-agonist, NIZ985. Importantly, both exercise and NIZ985 increase the therapeutic sensitivity of intractable murine pancreatic tumors (21). For the development of physical activity programs for patients on discharge from hospital, it is important to learn about the current status of physical activity levels in survivors after pancreaticoduodenectomy and their perioperative influences.

We quantified exercise among cancer survivors following pancreaticoduodenectomy in this study. To the best of our knowledge, this is the first study that has determined the predictors of physical activity level in cancer survivors after pancreaticoduodenectomy in China. Only 16.6% of participants reported performing aerobic exercise meeting ACSM Exercise Guidelines for cancer survivors. This proportion was lower than that of a study among Americans (15). Westerners are more physically active than Chinese (22). Because of the traditional Chinese belief of the need for more rest and recuperation after illness, the median sedentary time in our study was 7 (5-8) h, which was 1 h higher than the average for the Chinese population in a multi-country census (23). The inactive prevalence and high sedentary time prevalence are consistent with the consensus of previously published Chinese physical activity guidelines (24). As guideline adherence to physical activity following cancer treatment is associated with improved health-related outcomes, it is recommended that healthcare professionals should strengthen the physical activity task to improve the physical activity level of cancer survivors after pancreaticoduodenectomy on the basis of each individual’s health status.

In this study, we found that preoperative nutritional risk, BMI, operation duration, pancreatic leakage, abdominal bleeding, and time between surgery and survey were associated with physical activity level. The results are partially consistent with Parker et al.’s study, who found that age, BMI, a history of pancreatoduodenectomy or total pancreatectomy, barrier self-efficacy, and behavioral regulation were associated with physical activity (15). However, there is no difference in physical activity between the elderly and non-elderly pancreatic cancer survivors in our study. We further reviewed the literature on differences in physical activity levels by age in China and the United States. According to the 2020 National Fitness Activity Status Survey Bulletin released by the China National Physical Fitness Monitoring Center, the proportion of adults regularly participating in physical activity was 30.3%, with the highest proportion of 31.7% in the 40–49 age group, and the proportion of older persons regularly participating in physical activity was 26.1% (25). However, Varma found that the total physical activity levels of elderly Americans are lower than people aged 19–59 years old. Specifically, total physical activity levels at age 19 are comparable to levels at age 60. During young adulthood (ages 20–30), total and light intensity physical activity increases by age and then stabilizes during midlife (ages 31–59) partially due to an earlier initiation of morning physical activity (26).

Our results indicate that operation duration, pancreatic leakage, and months between pancreaticoduodenectomy and survey completion were the potential risk factors of survival physical activity for cancer patients after pancreaticoduodenectomy. For one-unit increase in operation duration, there is approximately a 0.5% reduction in the level of active physical activity participation among cancer survivors after pancreaticoduodenectomy. Hogenbirk et al. found that operation duration was associated with postoperative physical activity in patients with cancer who underwent major abdominal cancer surgery (27). Prolonged surgery means a longer duration of general anesthesia and stress response, which may increase the risk of complications and affect metabolism and the immune system. A meta-analysis confirmed that the likelihood of complications increased significantly with prolonged operation duration, approximately doubling with operative time thresholds exceeding 2 or more hours, and a 14% increase in the likelihood of complications for every 30 min of additional operating time (28). Shen et al. verified that operation duration is an independent risk factor for patients’ short-term and long-term outcome after radical colorectal surgery (29). However, we did not find studies focus on the operation duration on physical activity in cancer survival time. More research is needed to further clarify this association between physical activity levels and operation duration in cancer survivors.

We found that patients without pancreatic leaks were 2.690 times more likely to perform active physical activity in survival than those with pancreatic leakage. Cancer itself and the side effects associated with cancer treatment are the biggest barriers to physical activity for patients with cancer (30). Postoperative pancreatic fistula is the most serious complication after pancreaticoduodenectomy. Pancreatic cancer survivors have a high frequency of pancreatic insufficiency after surgery, and postoperative pancreatic fistula increases the risk of pancreatic exocrine insufficiency. Kanwat found that patients with severe exocrine insufficiency had a higher fatigue score than those without (31), while fatigue was the most frequently reported barrier to exercise in patients with cancer (32).

Our results showed that for each unit increase in follow-up time, patients after pancreaticoduodenectomy were 1.057 times more likely to participate in active physical activity. Months between cancer survivor surgical tumor resection and survey completion ranges from 16 to 45 months in our study, and we found that during this phase, patients’ physical activity levels increased as the time after surgery increased. The result was consistent with the findings of Fang et al., which found that the proportion of physical activity levels increased with time in postoperative patients with breast cancer (33). A similar result was seen in the study by Zhou et al., who found that a postoperative period of less than 1 year was an independent risk factor for inactivity in patients with colorectal cancer stoma (34). Cancer survivors have significantly lower levels of physical activity while undergoing anticancer treatment, but patients’ exercise levels recover somewhat after treatment.

To our knowledge, this is the first article on physical activity levels in Chinese cancer survivors after pancreaticoduodenectomy. However, the study has some limitations. Firstly, because of the large number of non-responders to the interview, there is a potential reporting bias. Secondly, we did not evaluate participants’ exercise motivation and exercise barrier self-efficacy, which may have an intrinsic influence on the patient’s physical activity level. Thirdly, we did not analyze the severity grades of postoperative complications after pancreaticoduodenectomy; thus, the impact of severe complications on the physical activity level of patients with cancer after PD remains to be studied. Fourthly, when analyzing the number of hospitalizations, we only analyzed the number of hospitalizations at our center, which may lead to bias in the results. In addition, we fail to analyze the correlation of current health and physical status with physical activity status. Finally, we did not list adverse events after adjuvant chemotherapy and the postoperative complication grading, which may affect the postoperative activity level.

## Conclusion

We quantified physical activity among cancer survivors after pancreaticoduodenectomy. We found that half of the cancer survivors experienced active physical activity, but only a small percentage of individuals met the guideline-recommended level of aerobic exercise. Operation duration, postoperative pancreatic fistula complication, and time after surgery were related to the level of physical activity in survivors after pancreaticoduodenectomy. More physical activity support should be provided to patients after pancreaticoduodenectomy, and operation duration, postoperative pancreatic fistula, as well as follow-up time should be taken into consideration when giving exercise instructions to postoperative survivors of pancreaticoduodenectomy.

## Data Availability

The raw data supporting the conclusions of this article will be made available by the authors, without undue reservation.
